# Real-world survival of US patients with intermediate- to high-risk myelofibrosis: impact of ruxolitinib approval

**DOI:** 10.1007/s00277-021-04682-x

**Published:** 2021-10-09

**Authors:** Srdan Verstovsek, Shreekant Parasuraman, Jingbo Yu, Anne Shah, Shambhavi Kumar, Ann Xi, Claire Harrison

**Affiliations:** 1 Department of Leukemia, The University of Texas MD Anderson C39.8% were male. Among patients withancer Center, 1515 Holcombe Blvd, Houston, TX 77030 USA; 2 Incyte Corporation, Wilmington, DE USA; 3 Avalere Health, Washington, DC USA; 4 Guy’s and St. Thomas’ NHS Foundation Trust, Guy’s Hospital, London, UK

**Keywords:** Medicare Fee-for-Service; Myelofibrosis; Real world; Ruxolitinib; Survival

## Abstract

The Janus kinase inhibitor ruxolitinib is approved for the treatment of myelofibrosis (MF) and improved overall survival (OS) versus control therapy in the phase 3 COMFORT trials. The aim of this retrospective analysis was to examine the real-world impact of ruxolitinib on OS in patients with MF. The US Medicare Fee-for-Service claims database (parts A/B/D) was used to identify patients with ≥ 1 inpatient or ≥ 2 outpatient claims with an MF diagnosis (January 2010–December 2017). Eligible patients with MF were ≥ 65 years old (intermediate-1 or higher risk based on age). Patients were divided into 3 groups based on ruxolitinib approval status at diagnosis and ruxolitinib exposure: (1) preapproval, ruxolitinib-unexposed; (2) post-approval, ruxolitinib-unexposed; and (3) post-approval, ruxolitinib-exposed. In total, 1677 patients with MF were included (preapproval [all ruxolitinib-unexposed], *n* = 278; post-approval, *n* = 1399 [ruxolitinib-unexposed, *n* = 1127; ruxolitinib-exposed, *n* = 272]). Overall, median age was 78 years, and 39.8% were male. Among patients with valid death dates (preapproval, *n* = 119 [42.8%]; post-approval, ruxolitinib-unexposed, *n* = 382 [33.9%]; post-approval ruxolitinib-exposed, *n* = 54 [19.9%]), 1-year survival rates were 55.6%, 72.5%, and 82.3%, and median OS was 13.2 months, 44.4 months, and not reached, respectively. Risk of mortality was significantly lower post- versus preapproval regardless of exposure to ruxolitinib (ruxolitinib-unexposed: adjusted hazard ratio [HR], 0.67; ruxolitinib-exposed: adjusted HR, 0.36; *P* < 0.001 for both); post-approval, mortality risk was significantly lower in ruxolitinib-exposed versus ruxolitinib-unexposed patients (adjusted HR, 0.61; *P* = 0.002). Findings from this study complement clinical data of ruxolitinib in MF by demonstrating a survival benefit in a real-world setting.

## Introduction

Myelofibrosis (MF) is a chronic myeloproliferative neoplasm characterized by hyperproliferation of myeloid cells, bone marrow fibrosis, and burdensome constitutional symptoms [1–3]. MF can develop de novo (primary MF) or may occur in patients with antecedent polycythemia vera (PV) or essential thrombocythemia (ET; secondary MF) [1]. Patients with MF have reduced overall survival (OS) compared with the general population [4, 5]. Based on recent population-based studies, the estimated incidence of primary MF in the USA is approximately 0.3 per 100,000 person-years and median OS is < 5 years from the time of diagnosis [6, 7]. When stratified by risk, median survival in patients with primary MF ranges from 2 years from diagnosis for high-risk MF to 11 years in low-risk MF by the International Prognostic Scoring System (IPSS) [8], which was designed to assess risk at the time of MF diagnosis.

Ruxolitinib, a Janus kinase (JAK) 1 and JAK2 inhibitor, was approved by the US Food and Drug Administration in November 2011 for the treatment of adult patients with intermediate- to high-risk primary or secondary MF based on data from the phase 3 COMFORT-I and COMFORT-II trials [9, 10]. Ruxolitinib significantly reduced spleen volume and improved MF-related symptoms and quality of life compared with placebo or best available therapy in the COMFORT studies [11, 12]. Furthermore, in the pooled analysis of COMFORT data, patients treated with ruxolitinib demonstrated improved OS compared with patients in the control arm (placebo, COMFORT-I; best available therapy, COMFORT-II; 5.3 versus 3.8 years, respectively; hazard ratio [HR], 0.70; 95% CI, 0.54–0.91; *P* = 0.007), and when patients were censored at crossover, the survival advantage was even greater (5.3 versus 2.4 years, respectively; HR, 0.53; 95% CI, 0.36–0.78; *P* = 0.001) [10]. In a phase 3b expanded access study (JUMP), reductions from baseline in spleen volume and MF symptom burden were observed in a large cohort of patients with MF receiving ruxolitinib treatment (*N* = 2233), including in patients ineligible for the COMFORT studies due to low-risk disease (i.e., low risk or intermediate-1) or low platelet count (i.e., < 100 × 10^9^/L) [13, 14]. Median OS for patients with MF enrolled in JUMP was 4.9 and 2.8 years among patients with intermediate-2 and high-risk MF, respectively [14].

Despite the established survival benefit of ruxolitinib in the clinical trial setting, limited real-world data exist. The objective of this analysis was to examine the real-world impact of ruxolitinib on patients with MF by comparing survival of patients newly diagnosed with MF before ruxolitinib approval with patients who were ruxolitinib-unexposed versus ruxolitinib-exposed after ruxolitinib approval.

## Methods

### Study design and patients

**Fig. 1:**
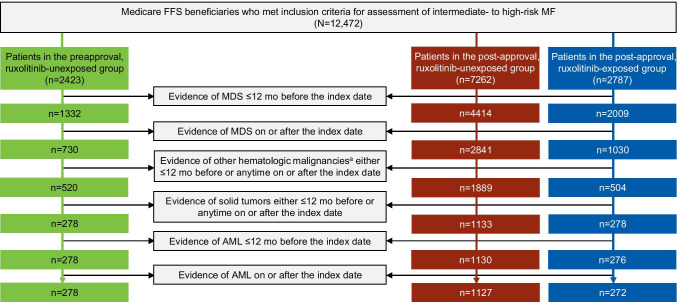
Patient attrition with exclusions. ^a^Excluding AML. AML, acute myeloid leukemia; FFS, Fee-for-Service; MDS, myelodysplastic syndrome; MF, myelofibrosis; RUX, ruxolitinib

A retrospective analysis was performed of the US Medicare Fee-for-Service claims database (parts A/B/D) to identify patients with ≥ 1 inpatient or ≥ 2 outpatient claims with a diagnosis of MF from January 2010 through December 2017. The index date was the date of MF diagnosis as indicated by the first qualifying MF claim. Eligible patients with MF were ≥ 65 years old (and therefore intermediate-1 or higher risk based on age) with a minimum of 12 months of pre-index continuous medical and pharmacy enrollment. Patients with evidence of MF diagnosis ≤ 12 months before the index date were excluded; additionally, patients with a diagnosis of myelodysplastic syndrome; other hematologic malignancies (i.e., leukemias, multiple myeloma, lymphomas); or solid tumors either ≤ 12 months before, on, or any time after index were excluded in a stepwise manner (Fig. 1).

Patients were divided into 3 groups based on ruxolitinib approval status at the time of diagnosis and subsequent patient exposure to ruxolitinib: (1) preapproval, ruxolitinib-unexposed (index year 2010–2011); (2) post-approval, ruxolitinib-unexposed (index year 2012–2017); and (3) post-approval, ruxolitinib-exposed (index year 2012–2017). Ruxolitinib exposure during the post-index period was determined based on patient prescription fill history; patients categorized as ruxolitinib-unexposed did not receive ruxolitinib at any time following the index event.

### Statistical analyses

Patient demographics and clinical characteristics were summarized using descriptive statistics. One- and 2-year survival rate and risk of mortality were estimated using Kaplan–Meier and Cox proportional hazards regression analyses, adjusting for baseline demographic and clinical characteristics. One-year mortality was evaluated from the index date through the end of 1 year. OS was evaluated from the index date through the entire length of follow-up until death or end of data availability. Patients without a death date were censored at disenrollment or at the end of study period, whichever occurred first.

## Results

### Baseline patient characteristics

The analysis included 1677 eligible patients with an MF diagnosis, with 278 patients diagnosed pre-ruxolitinib approval (all ruxolitinib-unexposed) and 1399 diagnosed post-ruxolitinib approval (ruxolitinib-unexposed, *n* = 1127; ruxolitinib-exposed, *n* = 272; Fig. 1). Overall, median age was 78 years, 39.8% of patients were male, and 84.1% were White. The preapproval group was the oldest (median age, 80.7 years) and had the highest proportion of female patients (70.1%; Table 1). History of PV and ET also varied among the 3 groups, with the highest proportion of patients with previous history in the post-approval ruxolitinib-exposed group (PV, 20.2%; ET, 19.5%). The Charlson Comorbidity Index was lowest among post-approval ruxolitinib-exposed patients (mean [SD], 2.2 [2.2] versus 3.7 [2.7] for the preapproval group and 3.2 [2.9] for the post-approval ruxolitinib-unexposed group). Median duration of follow-up was 12.5 months for the preapproval group, 10.2 months for post-approval ruxolitinib-unexposed group, and 14.0 months for the post-approval ruxolitinib-exposed group.

**Table 1:** Patient demographics and clinical characteristics at diagnosis

Characteristic	Preapproval ruxolitinib-unexposed (*n* = 278)	Post-approval ruxolitinib-unexposed (*n* = 1127)	Post-approval ruxolitinib-exposed (*n* = 272)
Age, years, median (range)	80.7 (65–102)	78.0 (65–105)	75.4 (65–94)
Sex, *n* (%)			
Female	195 (70.1)	662 (58.7)	152 (55.9)
Male	83 (29.9)	465 (41.3)	120 (44.1)
Race, *n* (%)			
White	236 (84.9)	934 (82.9)	240 (88.2)
Black	24 (8.6)	115 (10.2)	13 (4.8)
Other/unknown	18 (6.5)	78 (6.9)	19 (7.0)
Geographic region (%)			
South	101 (36.3)	405 (35.9)	98 (36.0)
idwest	78 (28.1)	252 (22.4)	65 (23.9)
Northeast	54 (19.4)	254 (22.5)	51 (18.8)
West	43 (15.5)	214 (19.0)	58 (21.3)
History of PV, *n* (%)	34 (12.2)	77 (6.8)	55 (20.2)
History of ET, *n* (%)	42 (15.1)	179 (15.9)	53 (19.5)
Charlson Comorbidity Index, mean (SD)	3.7 (2.7)	3.2 (2.9)	2.2 (2.2)
Duration of follow-up, median, months	12.5	10.2	14.0

*ET*, essential thrombocythemia; *PV*, polycythemia vera; *RUX*, ruxolitinib

### Overall survival

**Fig. 2:**
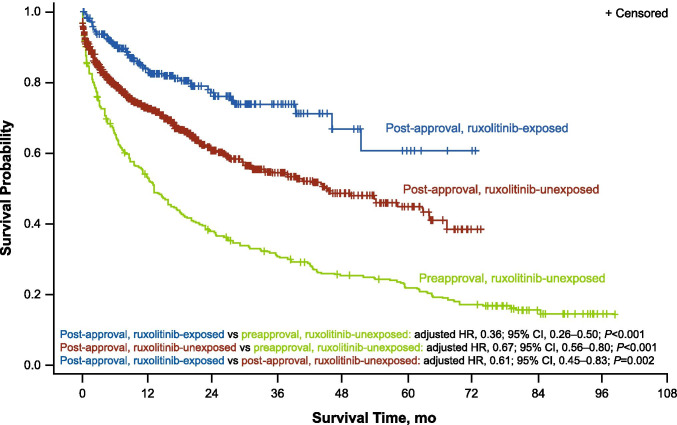
OS for patients newly diagnosed with intermediate- to high-risk MF. One-year survival rate and risk of mortality estimated using Kaplan–Meier and Cox proportional hazards regression analyses. HR, hazard ratio; MF, myelofibrosis; OS, overall survival

Valid death dates were available for 119 (42.8%) patients in the pre-ruxolitinib approval group and 436 (31.2%) in the post-ruxolitinib approval group (ruxolitinib-unexposed, *n* = 382 [33.9%]; ruxolitinib-exposed, *n* = 54 [19.9%]; Table 2). Among patients with valid death dates, the 1-year survival rate (95% CI) was 55.6% (49.4–61.3%) for the pre-ruxolitinib approval group, 72.5% (69.5–75.2%) for the post-approval ruxolitinib-unexposed group, and 82.3% (76.7–86.7%) for the post-approval ruxolitinib-exposed group. Median (95% CI) OS was 13.2 months (10.2–17.1) for the pre-ruxolitinib approval group, 44.4 months (37.3–62.0) for post-approval ruxolitinib-unexposed patients, and not reached (51.0–not reached) for post-approval ruxolitinib-exposed patients. Compared with the pre-ruxolitinib approval group, risk of mortality was significantly lower in the post-approval groups regardless of exposure to ruxolitinib (ruxolitinib-unexposed: adjusted HR, 0.67; 95% CI, 0.56–0.80; *P* < 0.001; ruxolitinib-exposed: adjusted HR, 0.36; 95% CI, 0.26–0.50; *P* < 0.001; Fig. 2). Among the patients in the post-approval groups, those who were exposed to ruxolitinib had a significantly lower risk of mortality than those who were never exposed to ruxolitinib (HR, 0.61; 95% CI, 0.45–0.83; *P* = 0.002).

**Table 2:** Survival outcomes in the post-approval time frame

	Post-approval ruxolitinib-unexposed (*n* = 1127)	Post-approval ruxolitinib-exposed (*n* = 272)
Patients with valid death dates, *n* (%)	382 (33.9)	54 (19.9)
Median OS (95% CI), months	44.4 (37.3–62.0)	NR (51.0–NR)
1-year survival rate (95% CI), %	72.5 (69.5–75.2)	82.3 (76.7–86.7)
2-year survival rate (95% CI), %	60.6 (56.9–64.0)	76.1 (69.2–81.7)

*NR*, not reached; *OS*, overall survival

## Discussion

In this real-world study of US patients diagnosed with intermediate- to high-risk MF, OS was significantly longer for patients who received ruxolitinib than for those who did not receive ruxolitinib, providing real-world data to complement the survival benefit observed in the COMFORT studies. Few other real-world studies have evaluated the effect of ruxolitinib or diagnosis period (i.e., before or after ruxolitinib approval) on survival in patients with MF. A study from Sweden and Norway reported that the OS rate of patients with MF treated with ruxolitinib was estimated at 80% at 1 year and 52% at 4 years [15]. A study from Turkey reported 1- and 3-year OS estimates after ruxolitinib treatment initiation of 90% and 72%, respectively [16]. A single-institution retrospective analysis of medical charts from patients with MF demonstrated improved survival among patients with myelofibrosis since 2010 compared with patients diagnosed prior to 2010 (HR [95% CI], 0.7 [0.59–0.82]; *P* < 0.001) [17]; patients treated with ruxolitinib had superior survival to those who did not receive ruxolitinib irrespective of diagnosis time period. In contrast, another study of population-based registry data from the USA showed no improvement in survival among patients with primary MF diagnosed pre-ruxolitinib approval (2007–2011; 4-year relative survival, 55%) versus post-ruxolitinib approval (2012–2016; 4-year relative survival, 56%) [18]. Of note, ruxolitinib exposure was not captured in that study and survival data post-2016 were not reported; furthermore, only patients with primary MF were included, and risk status was not provided.

In the current study, OS was longer among patients diagnosed after ruxolitinib approval compared with those diagnosed before ruxolitinib approval. Many factors may have contributed to this observed improvement, including increased disease awareness and improved patient management for individuals with MF over time. Within the time frame of this study, 2010–2017, guidelines for the management of MF became available from the European LeukemiaNet in 2011 [19], the World Health Organization updated their guidelines in 2016 (last revised in 2008) [20], and the National Comprehensive Cancer Network® (NCCN®) Guidelines for Myeloproliferative Neoplasms became available in 2017 [21]. In addition, risk stratification tools for MF have expanded, and their uses have been refined over the years [22]. Prognostic modeling for MF started with the development of IPSS in 2009, which categorized risk as low, intermediate-1, intermediate-2, or high using factors such as age, constitutional symptoms, and blood counts [8]. Currently, NCCN® guidelines recommend stratification of patients into higher or lower risk, using scores from Mutation-Enhanced IPSS (MIPSS-70) or MIPSS-70 + version 2.0 (preferred), Dynamic IPSS (DIPSS)-Plus (if molecular testing is not available), DIPSS (if karyotyping is not available), or MF Secondary to PV and ET-Prognostic Model (MYSEC-PM) [23]. These newer prognostic systems take into account additional factors, such as molecular testing results, transfusion dependence, and karyotype [2], which may contribute to improved disease management compared with the IPSS.

The limitations of this study are consistent with the retrospective nature of real-world claims-based analyses. For example, some clinical characteristics (e.g., hemoglobin level, circulating blast percentage, symptoms) were not available in the claims database. Furthermore, no comparisons of patient characteristics were performed at index, so differences between groups may have contributed to observed outcomes.

In conclusion, this real-world study demonstrated improved survival among patients with MF in the years since ruxolitinib approval in 2011. Patients who were exposed to ruxolitinib had the best survival outcomes of the studied cohorts. The findings generally complement those from clinical studies demonstrating a survival benefit with ruxolitinib; however, additional real-world studies are needed to better understand the impact of ruxolitinib on survival among patients with MF.

## Data Availability

The data described in this paper are sourced from Centers for Medicare and Medicaid Services (CMS) Medicare FFS claims and enrollment data. The analytic file constructed for this analysis cannot be shared due to restrictions set forth in the governing Data Use Agreement with CMS. Researchers may request use of CMS data through the Research Data Assistance Center.
